# Epistemic Curiosity in Kea Parrots and Human Children

**DOI:** 10.1162/OPMI.a.34

**Published:** 2025-09-17

**Authors:** Gabriella E. Smith, Megan L. Lambert, Eliza Swindell, Jan M. Engelmann, Christoph J. Völter

**Affiliations:** Comparative Cognition, Messerli Research Institute, University of Veterinary Medicine Vienna, Medical University of Vienna, University of Vienna, Vienna, Austria; School of Psychology and Neuroscience, University of St. Andrews, St. Andrews, UK; Department of Psychology, University of California, Berkeley, Berkeley, CA, USA; Department of Comparative Cultural Psychology, Max Planck Institute for Evolutionary Anthropology, Leipzig, Germany

**Keywords:** epistemic curiosity, information seeking, violation-of-expectation, kea parrot, children, curiosity

## Abstract

Both human children and animals seek information following a violation-of-expectation event, but little research suggests the latter do so for the sake of it. In this preregistered experiment, we compared epistemic curiosity—the pursuit of information for its own sake—in kea parrots (*Nestor notabilis*) and three-year-old human children (*Homo sapiens*) following a violation-of-expectation event. Subjects were trained to push a tool into an apparatus that produced a reward before the apparatus was surreptitiously made non-functional in following trials. In both functional and non-functional trials, after solving the task, subjects were rewarded and allowed to explore the apparatus for thirty seconds with the opportunity to peek into the side of the apparatus. We found that relatively more kea peeked than children, but the children and not the kea were significantly more likely to peek in the non-functional versus functional trials, particularly when the researcher was absent. While both species showed markers of curiosity in the experiment, we found expectancy-violation-induced epistemic curiosity only in the children and not the kea in this context.

## INTRODUCTION

Curiosity exists on many spectra. Defined broadly as the pursuit of information (Berlyne, 1949, 1950, 1954, 1960; Kidd & Hayden, 2015; Loewenstein, 1994), the characteristics of curiosity can depend on the interactions among a variety of variables, including but not limited to: the individual [e.g., neotic style (response to novelty, Corey, 1978); age; sex; experience; etc., (see Pisula, 2009)]; the environment (e.g., captivity vs. the wild; Forss et al., 2022); presence of conspecifics (Damerius, Graber, et al., 2017); ecological variation (Schuppli et al., 2023); stress (Rosellini & Widman, 1989); and the stimulus (e.g., novelty/surprisingness; complexity/salience; species relevance/importance; Berlyne, 1966; Coleman & Wilson, 1998; Markey & Loewenstein, 2014) (see Ajuwon et al., 2025; Forss et al., 2024 and Poli et al., 2024 for recent reviews). At a basal level, curiosity can manifest as the simple pursuit of novelty (James, 1890), such as spatial exploration of a novel environment (e.g., Dember, 1956), termed by Berlyne (1954) as perceptual curiosity. Curiosity can also manifest at an abstract level, such as the pursuit of information for information’s sake, known as epistemic curiosity (Berlyne, 1954; Day, 1971; Loewenstein, 1994).

Epistemic curiosity has been thoroughly examined in humans but less so in nonhuman animals (hereafter, animals; Völter et al., 2020). Specifically, epistemic curiosity is characterized by non-extrinsically rewarded investigative behaviors, such as infants looking longer at, and preferentially exploring surprising events (e.g., ball passing through a solid wall) relative to less surprising events (e.g., ball not passing through the wall; Perez & Feigenson, 2022). Infants aged 11–16 months even actively test hypotheses corresponding with expectations violated in a form of epistemic curiosity called explanation seeking (e.g., dropping objects that appear to float horizontally off a shelf rather than fall to the ground; Stahl & Feigenson, 2015; testing a new toy and/or appealing to a parent following failure to activate a toy; Gweon & Schulz, 2011). As seen here, the best cases to observe epistemic curiosity are likely those in which there is no change in corresponding external reward and thus true pursuit of information for information’s sake.

Cases of epistemic curiosity in animals are more rare. Epistemic curiosity might be seen as violating the golden rule of homeostasis which suggests animals do not waste time or energy on behavior that does not serve reproductive or survival purposes (Cannon, 1932). While the presence of epistemic curiosity in animals has proven hard to document, there are a few putative cases. In an experiment studying kakariki parrots (*Cyanoramphus novaezelandiae*), subjects were found to be more likely to change their exploration when a familiar object’s center-of-gravity had been altered (i.e., addition of a loose object inside) compared to other change conditions (e.g., color, shape; Demery, 2013). A potential caveat to this study, however, is that objects did not hold a functional role in the parrot subjects’ environment. Thus, this difference in exploration may have been rooted simply in preference, rather than investigation. This confound was considered in a recent study by Völter et al. (2023) in which dogs (*Canis familiaris*) were shown a ball toy that either violated or behaved in accordance with continuity expectations. The authors then examined how the dogs played with the toy after this demonstration. Those dogs that had been presented with the violation-of-expectation event played with the toy more than those that had been presented with the predictable event. It is unclear, however, whether this was due to heightened attention following the violation of expectation or actual epistemic curiosity.

Similar to the developmental psychology research above, the epistemic phenomenon of explanation seeking has been examined in animals, but in just one study. Povinelli and Dunphy-Lelii (2001) trained chimpanzees (*Pan troglodytes*) and five-year-old human children to upright a block for a reward before being given sham blocks that could not stand upright as before (Exp. 1: beveled top/bottom; Exp. 2: shifted center-of-gravity). Although both species were found to explore the sham blocks more in Experiment 1, the authors stated that the children and not the chimpanzees expressed explanation seeking. They based this conclusion on the finding that in Experiment 2 (with visually identical sham and training blocks) only children but not chimpanzees performed more investigative behaviors (e.g., visual and tactile inspections) of the sham blocks (compared to the trained blocks). The authors thus asserted that humans, and not animals, express explanation seeking (Povinelli & Dunphy-Lelii, 2001). What is important to note, however, is that since the sham blocks could virtually never be uprighted, the corresponding food rewards were withheld in this condition. Thus, the disparity in reward violates the definition of epistemic curiosity, and thus makes its presence/absence difficult to interpret.

The current study was designed to tackle the issues of reward consistency, object functionality, and increased attention by investigating epistemic curiosity in both children and animals trained on a tool-use mechanism to acquire a reward in both the functional and non-functional conditions. The animal species studied here were kea parrots (*Nestor notabilis*), chosen not only for their curiosity (Diamond & Bond, 1999), but also extensively documented play, exploratory behaviors and problem-solving abilities (Auersperg et al., 2011; Gajdon et al., 2013; Huber & Gajdon, 2006; Lambert et al., 2017) often compared to those of human children (Huber & Gajdon, 2006; Range et al., 2009). The age of three years in the child participant were chosen for this study for two reasons: first, the onset of epistemic curiosity is theorized to take place around three years-old (e.g., explanation seeking; Liquin & Lombrozo, 2020; but see Stahl & Feigenson, 2015); and piloting with two-year-old children in this project revealed that they had difficulty operating the apparatus. Also note that juvenile kea were not tested in this study due to there being only three in the flock at the time, and they were not yet experienced with testing procedures. From an evolutionary perspective, comparing humans and kea offers valuable information into how epistemic curiosity manifests and potentially evolved convergently in two highly curious species separated by around 319 million years (Kumar et al., 2022).

In this study, both the kea and children were trained to push a block into a mechanism, that when functional, released a reward (functional condition). However, when the mechanism was surreptitiously made non-functional in later trials, the experimenter delivered the reward instead (non-functional condition). In both conditions, subjects were given thirty seconds after they had received the reward to interact with the mechanism at-will, with the option to peek inside a non-occluded portion of the mechanism as an expression of expectancy-violation-induced epistemic curiosity. We hypothesized that both species would exhibit epistemic curiosity when the apparatus failed to function, peeking inside the mechanism more often in non-functional trials versus functional trials.

## EXPERIMENT 1: KEA

### Materials and Methods

#### Subjects.

This within-subjects experiment studied the epistemic curiosity behavior of 17 captive, adult kea parrots (8F, 9M; age range of 5–24 years; average age of ∼13 years-old; age standard deviation of ∼5 years; Table S1 in Supplementary Materials) kept in a large outdoor aviary (52 × 10 × 4 m) at Haidlhof Research Station in Bad Vöslau, Austria. From September to December 2022, individual birds were tested weekly in visually isolated testing compartments (6 × 10 × 4 m), to which they are already habituated. As is procedure, subjects participated voluntarily in the experiment by entering the testing compartment on their own. If, once in the testing compartment, an individual did not appear attentive to the experiment for more than thirty seconds (e.g., running around; waiting by the compartment door), they were considered uninterested in participating and were thus released back into the aviary. Parrots are maintained on a daily diet of seeds, fruits, vegetables, and meat, with access to water ad libitum (also during testing).

#### Apparatus.

**Figure 1.**
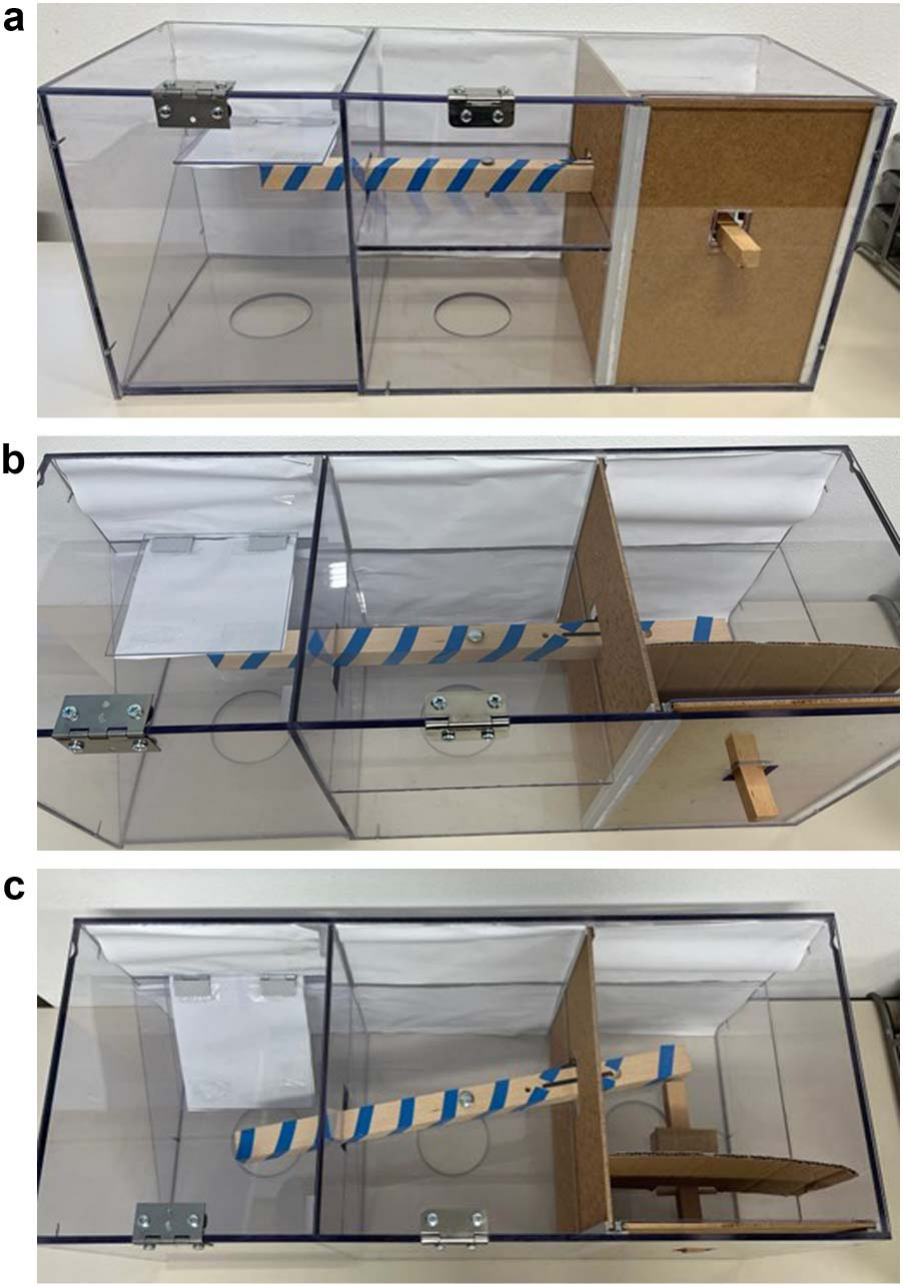
Testing apparatus **a.** front view, **b.** top view (unsolved), and **c.** top view (solved). Parrots could push the wooden stick into the right compartment, causing the arm to move, thereby dropping the tray in the left compartment and releasing a peanut from a small front slot at the bottom of the left compartment. The right side of the box could be occluded (shown here) or non-occluded.

The apparatus used in this experiment was a Plexiglas box (75 × 30 × 33 cm) divided into three compartments designed to separate the problem-solving action (right) from the location the peanut was dispensed from (left) (Figure 1). Removable lids on top of the apparatus compartments allowed for insertion of occluder materials (right) and placement of the peanut on the tray (left). A wooden arm inside (50 × 3 × 3 cm), wrapped with blue tape for visual saliency ran the length of the apparatus. Importantly, a tray in the leftmost compartment onto which a peanut would be placed balanced on top of the arm, so that when a bird solved the task in a functional trial, the arm was pushed out from under the tray, causing the peanut to fall. To switch between functional and non-functional trials, the researcher could remove a blocker inside the hinge so that when the arm was pushed in non-functional trials, the part balancing the tray remained unmoved. A non-removable tool (17.5 × 2 × 2 cm) in the right compartment could be pushed to slide the arm out from underneath the tray and cause the peanut to fall through an opening at the bottom of the left compartment (functional trial). Importantly, in non-functional trials, removing the blocker inside the hinge divided the arm into two parts and caused the part on the left side to be immovable, thus preventing the tray from dropping. In both functional and non-functional trials, a thick cardboard occluder was placed in a slot on the front of the right compartment so that if a bird wanted to peek, it could do so on the transparent right side. Opaque white paper was applied on the back of the apparatus in all trials, as well as underneath the tray for visual saliency.

#### Procedure.

##### Training.

Birds were trained individually in one of the two testing compartments of the aviary (areas temporarily cordoned off by mesh and doors during testing). Importantly, testing compartments can be further split in two by mesh, creating one compartment where a bird waits (the “waiting compartment”), and another where they engage with the experiment. During training, birds were tasked with pushing the tool into the non-occluded apparatus, thus shifting the arm out from under the tray and releasing the peanut on top of it. Birds first received a familiarization session. In this session, with a bird in the waiting compartment, the experimenter lifted the left lid of the apparatus and baited the tray with a peanut quarter, closed the lid, and called the subject into the compartment with the apparatus, and then allowed him/her to familiarize themselves with the apparatus for five minutes. Parrots that did not spontaneously and successfully push in the tool and release the peanut in this session then underwent a shaping procedure to ensure that they could push the block into the apparatus (see Supplementary Materials). After the first successful solve (either spontaneously or after shaping), they moved on to criterion trials. Birds could move on to the experiment once they reached the criterion of five consecutive successful solves (releasing peanut in three minutes) out of six trials. While the study began with 19 subjects, two did not pass the training phase due to disinterest in the task. On average, the 17 remaining subjects took three days to master solving the apparatus (minimum of 1 day; maximum of 6 days) before moving onto the experiment.

##### Experimental Trials.

Experimental trials were also conducted in one of the two testing compartments of the aviary, and involved eight sessions of five consecutive trials in the following order: three refresher trials (identical to training and without occluder); one functional trial and one non-functional trial (both with occluder), the order of the last two counterbalanced within and between individuals. Refresher trials resembled training in that after the tray was baited, individual birds were called into the compartment with the apparatus and allowed to solve the (non-occluded) task within three minutes. In the last two trials, the experimenter occluded the front right side, baited the tray, and then lifted the right lid and pretended to remove the blocker inside the hinge (functional trial) or removed the blocker inside the hinge (non-functional trial). Importantly, due to the vantage from the waiting compartment, subjects could observe the researcher interacting with the apparatus (thus requiring the preparation actions to be identical), but could not see exactly how the apparatus was prepared specifically in each trial type due to the occluders at the back of the apparatus. After calling in the bird, the experimenter either allowed the subject to solve (functional trial) or attempt to solve for the peanut (non-functional trial). In functional trials, after the subject solved for the peanut (which landed on the sand immediately after the solve), the experimenter walked over to the apparatus, lifted up the left lid and pretended to place a peanut on the sand, and then closed the left lid again. The kea took on average 8.0 seconds (se: ± 0.9) to solve for the peanut once released from the waiting compartment, and could see and hear the platform collapse and release the peanut during functional trials, consistent with their experience during the familiarization and refresher trials. In both trials, functional and non-functional, the kea could potentially hear the peanut land on the sand, and they could clearly see it as they were swift to eat it. Birds waited around 30–100 seconds in the waiting compartment between trials as the experimenter reset and prepared the apparatus, this variation in duration affected by the tape used to secure the occluders as it often needed to be reapplied.

In non-functional trials, after the subject attempted (and failed) to solve for the peanut, the experimenter walked over, lifted up the left lid of the apparatus, placed the peanut on the sand (as quickly as possible to resemble the timing of functional trials), and closed the left lid again. In both functional and non-functional trials, birds could immediately access and eat the reward as soon as the tool was inserted to the apparatus consistent with training, either by the tray falling (functional trial), or being placed on the ground beneath the tray by the experimenter (non-functional trial).

**Figure 2.**
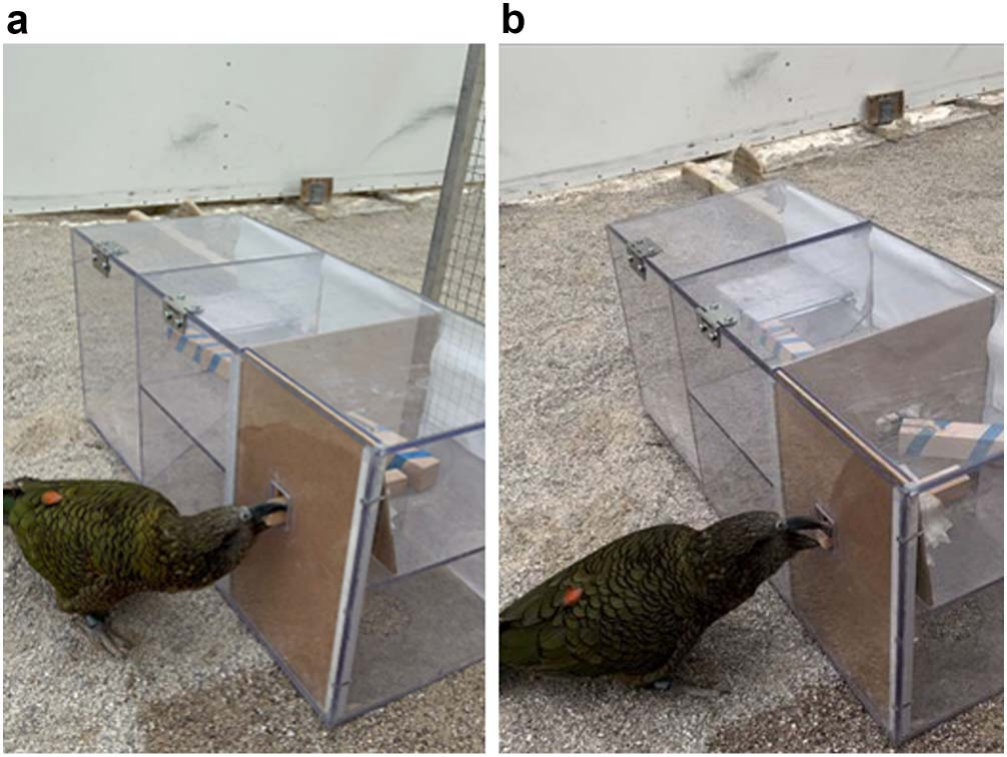
Kea subject performing **a.** functional trial, and **b.** non-functional trial. In non-functional trials, the removal of the blocker inside the hinge in the arm rendered the arm non-functional.

Importantly, consistency in the researcher’s actions between conditions (e.g., hinge blocker removal; peanut placement) was critical so as to discern any peeking as due to the apparatus rather than the researcher’s trial-based behavior. Furthermore, the placement of the peanut on the sand was kept identical in both functional and non-functional conditions so as to not cause any unintended conditional effects. In both conditions, subjects were allowed to explore for thirty seconds immediately after the delivery of the peanut before being ushered back into the waiting compartment (Figure 2). On average, each bird needed eight days to complete the eight experimental sessions, while the experiment as a whole took around 20 days to complete, lasting from September to December, 2022. All but two birds experienced one test session per day, these two birds accidentally tested twice in one day on just one occasion. One kea did participate in test sessions on consecutive calendar days, though this occurred only once. On average, birds experienced ∼3.7 calendar days between testing sessions.

#### Data Collection and Analysis.

##### Video Coding.

All experimental trials [272 trials (17 kea with 16 test trials each)] were video-recorded, and then later coded using BORIS. The interrater reliability (based on 18% of all test trials, *N* = 49) for binary peeking behavior variable was 0.77 (substantial agreement, Cohen’s kappa) and for the proportion peeking duration 0.87 (good reliability, ICC). All behaviors were coded as state behaviors, and peek counts, latencies and durations were coded as peeks into the right compartment of the apparatus either originating from the front, top, or side of the apparatus with the bird within one body length away. Importantly, the bird’s body could originate from any side, but their eye had to be parallel with a side in order to count it as a peek. Furthermore, while the front of the apparatus had been occluded to prevent peeking, during testing, some kea found and peeked into a small gap around the tool that had been imperceptible to the experimenters at the beginning of the experiment. Thus, these peeks were also included in the analysis as long as the bird was not manipulating the tool at this time.

##### Statistical Analysis.

The statistical computing software R (version 4.2.2; R Core Team, 2022) was used to examine binary, count, and proportional data of subjects’ behaviors during the thirty seconds of exploration following access to the peanut in experimental trials. Following our preregistered analysis plan (https://osf.io/gcnv9), we analyzed whether or not subjects peeked in any given trial (binary response), the number of peeks, and the proportion of time they spent peeking in the exploration period (relative to the trial duration). We analyzed these variables across all sessions as well as separately in just the first session. In an exploratory analysis, we also analyzed the latency to first peek (starting from time of reward arrival). We fitted generalized linear mixed models (GLMM) with binomial (binary data), Poisson (count data), or beta (proportion data) error structure as well as a linear mixed model (LMM) for the latency data (Bates et al., 2015; Brooks et al., 2017). We transformed the proportion data so that they did not comprise the extreme values 0 and 1, a requirement for fitting beta models (Smithson & Verkuilen, 2006). Following Smithson and Verkuilen (2006), we used the following transformation: y′ = [y(N − 1) + 1/2]/N to avoid zeros and ones.

The only test predictor was condition (two levels: functional and non-functional). We also included the following control predictor variables: the session number (1–8), the trial number of test conditions within sessions (1–2), and for the first session analysis, order of conditions (functional-first, non-functional-first). Additionally, we included the random intercept of subject ID and the random slopes of condition, trial, and session within subject ID where applicable. We checked for overdispersion (in the case of the beta and Poisson models) and collinearity and, in the case of the LMM, for homogeneous and normally distributed residuals. We used likelihood ratio tests (R function *drop1* with argument ‘test’ set to “Chisq”) with p-values smaller than .05 to draw inferences about fixed effects. An *a-priori* power analysis suggested a power of 66%–74% depending on the assumed effect size for the binomial GLMM (see preregistration for more details).

#### Ethical Statement and Preregistration.

The experiment was approved by the University of Veterinary Medicine Vienna's institutional ethics committee (ETK-119/06/2022) in accordance with Good Scientific Practice guidelines and national legislations. All subjects were housed in compliance with the Austrian Federal Act on the Protection of Animals (Animal Protection Act-TSchG, BGBl.I Nr.118/2004). Furthermore, since the study was non-invasive and focused solely on behavioral observations, it did not fall under the classification of an animal experiment as defined by the Austrian Animal Experiments Act (92, Federal Law Gazette No. 501/1989) and therefore did not require further permissions.

### Results

All 17 trained kea parrots finished the experiment, and 15 out of 17 subjects peeked at least once.

#### Confirmatory Analyses.

Peeking was examined during the exploration period, exploration in the first session, and during the exploration period as a proportion of the entire trial time. Regarding the former, we found no significant effects of *condition* (*χ*^2^(1) = 0.01, *p* = .942) (Figure 4a), *trial* (*χ*^2^(1) = 1.67, *p* = .197), nor *session* (*χ*^2^(1) = 1.71, *p* = .191) on binary peeking (Table S2 and Figure S1). Regarding the exploration period in just the first session, there was also no significant effect of *condition* (*χ*^2^(1) = 0.11, *p* = .742) nor *trial order* (*χ*^2^(1) = 2.77, *p* = .096) on binary peeking (Table S4).

When analyzing the exploration period as a proportion of trial time, we similarly found no significant effects of *condition* (*χ*^2^(1) = 0.02, *p* = .887) (Figure 6a), *trial* (*χ*^2^(1) = 1.1, *p* = .295), nor *session* (*χ*^2^(1) = 0.55, *p* = .457) on proportion peeking duration (Table S3). And when analyzing exploration period as a proportion of entire trial duration in the first session, we found no effect of *condition* (*χ*^2^(1) = 0.11, *p* = .743) nor *trial order* (*χ*^2^(1) = 1.93, *p* = .165) on proportion peeking duration (Table S5).

#### Exploratory Analyses.

With regards to the number of peeks per trial, we found no significant effects of *condition* (*χ*^2^(1) = 0.07, *p* = .787), *trial* (*χ*^2^(1) = 1.53, *p* = .216), nor *session* (*χ*^2^(1) = 1.92, *p* = .166) (Table S6). And regarding latency to the first peek in the exploration period, there was also no significant effects of *condition* (*χ*^2^(1) = 0.66, *p* = .418), *trial* (*χ*^2^(1) = 0.05, *p* = .823), nor *session* (*χ*^2^(1) = 0.07, *p* = .788) (Table S7).

## EXPERIMENT 2: CHILDREN

### Materials and Methods

#### Subjects.

Participants were 32, three-year-old children (*M* = 3.42 years, *SD* = 0.37, 17 girls). Participants identified as Asian (41.9%), White (19.3%), Hispanic or Latino (12.9%), multiple races (12.9%), Pacific Islander (6.4%), African or African American (3.2%), and other/unknown (3.2%) (see Table S8). Children were tested in schools and museums in the San Francisco Bay Area.

#### Apparatus.

**Figure 3.**
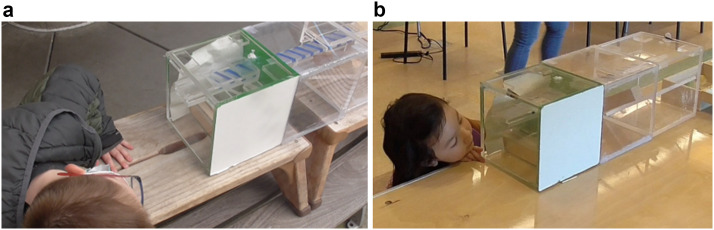
Examples of children peeking into the mechanism box during non-functional trials.

The same apparatus design was used between Experiment 1 and Experiment 2 (see Figure 3). There were minor differences in the dimensions of the box used with the children (60 × 20 × 20 cm) and the tool was removable from the box once it had been inserted.

#### Procedure.

##### Training.

Children received three training trials before the experimental trials began. In the first trial, Experimenter 1 (E1) would demonstrate how the apparatus functioned by pushing the wooden tool inside, causing a transparent ball capsule with a sticker inside to immediately fall out. The child would collect the ball and place it in a wooden box to the left of the apparatus at E1’s instruction (see Supplementary Materials for exact script and actions). In the next two trials, the child would independently push the tool to receive the newly replaced reward. If children were hesitant or needed guidance, E1 would provide aid. To avoid the child watching the researcher reset the apparatus, they were guided away from the apparatus by a second experimenter (E2) between trials.

##### Experimental Trials.

After the last training trial, children were guided back to the apparatus by E2, and each child received one test session comprising of four consecutive functional trials and four consecutive non-functional trials. The four functional trials always preceded the four non-functional trials to avoid a carryover effect from non-functional trials. In all eight trials, an occluder was placed on the front of the box’s right compartment. In the functional trials, children would insert the tool, and the sticker capsule would immediately fall into the opening of the left compartment. E1 would point to the opening, mirroring the actions of the non-functioning trials. Importantly, and similarly to the kea, children could see and hear the ball drop into the collection area of the apparatus, as well as see and hear the platform collapse.

In the non-functional trials, E1 would remove a small blocker in the arm to make the apparatus non-functional before a subject arrived. In these trials, children would insert the tool and the tray with the capsule would remain unmoved. E1 then swiftly lifted the lid of the left compartment and moved the sticker capsule from the tray to the opening. In both types of trials, after the child received the sticker capsule, E1 would then excuse themselves, leaving the child alone to interact with the apparatus at-will for thirty seconds. After the waiting period, E1 returned, and child was guided away from the apparatus by E2, as they had been in the training trials.

It is important to note methodological differences between experiments: While the kea experienced eight sessions of five trials (one functional, one non-functional per session), the children each experienced one session of eight trials (four functional, four non-functional). Furthermore, children and not kea experienced: a removable tool; verbal instruction of the apparatus; a slightly smaller apparatus; nonedible reward; and were left alone during the exploration period. All other critical aspects remained the same.

#### Data Collection and Analysis.

##### Video Coding.

Video recording and coding methods were identical to Experiment 1. Unlike the kea, peeking in the children was considered only if they occurred within six inches of the apparatus. Overall, there were 248 trials for the children (31 individuals with 8 test trials each). The interrater reliability (based on 22% of all test trials, *N* = 55) for binary peeking behavior variable was 0.87 (almost perfect agreement, Cohen’s kappa) and for the proportion peeking duration 0.98 (excellent reliability, ICC).

##### Statistical Analysis.

The analysis followed the preregistration (https://osf.io/68mzy/) and was similar to the one conducted on the kea data. We again analyzed the binary peeking response, the number of peeks, latency of first peeks (starting from time of reward arrival), and the proportion trial time spent peeking in the exploration period of experimental trials, and fitted the same models, but excluded session as a control predictor as children only participated in a single session. We had also planned to analyze whether the participants asked ‘why?’ questions, but this occurred too infrequently (see below), and so, in an exploratory analysis, we also analyzed whether or not children communicated at all with the experimenter during the trial. Note that separate analyses were conducted for peeking during the entire exploration period (beginning as soon as the apparatus was solved) as well as the time the researcher was absent to examine the effect of social influence on information seeking behavior. While the duration of time in which E1 was present following the solution varied across trials, on average, E1 was present after the solution for 9 seconds before leaving during the exploration period. We accounted for differences in the duration of the relevant periods by including the log-transformed durations as an offset term in the binomial and count models.

#### Ethical Statement.

The procedures were approved by the Committee for Protection of Human Subjects (CPHS) Institutional Review Board (IRB), protocol #2019-10-12605. Informed written consent was obtained from all parents of children and additional verbal assent was obtained from each child who participated in this study. Any identifiable image of a child in this manuscript was permitted by the parent.

### Results

Of the 32 children tested, 31 completed the experiment, as one child did not experience all trials due to experimenter error (see Table S8). Of these, 15 peeked at least once.

#### Confirmatory Analyses.

**Figure 4.**
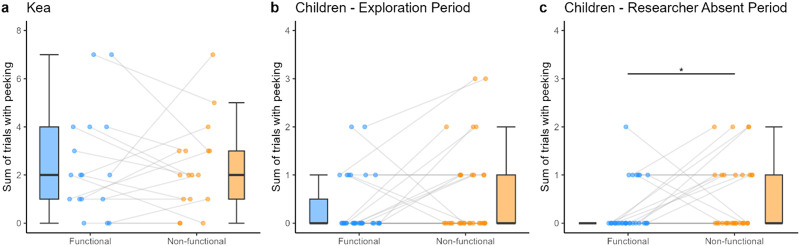
Box plots of the effects of condition on binary peeking behavior in **a.** kea in the exploration period, **b.** children in the exploration period overall, and **c.** in children while researcher was absent. A significant effect of condition on binary peeking behavior was found while the researcher was absent in children (based on the binomial GLMM, likelihood ratio test *p* < 0.05; Table S12). The horizontal line inside the box represents the median, the box the interquartile range, the whiskers the range excluding outliers. The dots represent mean individual values; same individuals are connected via the grey lines.

**Figure 5.**
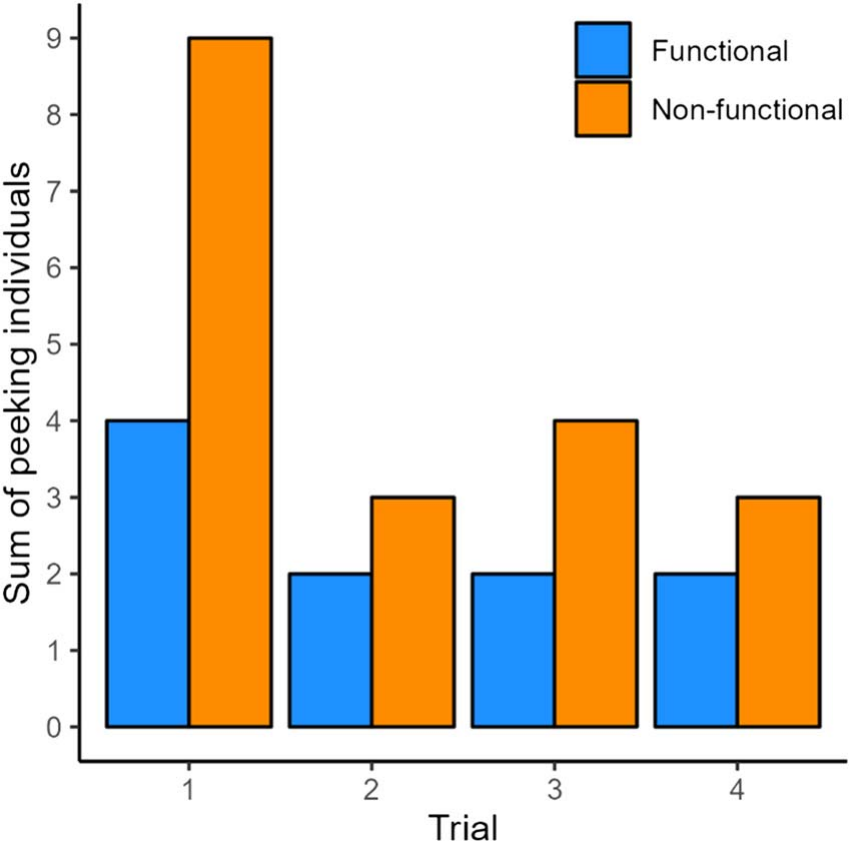
Bar plot showing the number of children peeking across trials in the functional and non-functional conditions. With increasing trial number, the children’s likelihood to peek decreased (based on a binomial GLMM, likelihood ratio test *p* < 0.05; Table S9).

**Figure 6.**
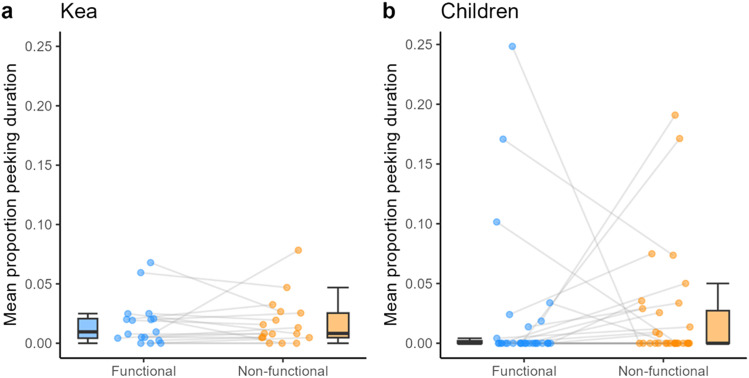
Box plots of the effects of condition on peeking duration in **a.** kea and **b.** children. No significant effects were found. The horizontal line inside the box represents the median, the box the interquartile range, the whiskers the range excluding outliers. The dots represent mean individual values; same individuals are connected via the grey lines.

Similarly to the kea data, peeking was examined both as binary variable and as proportion duration relative to the trial duration. Regarding just the exploration period (i.e., beginning as soon as the apparatus was solved), we found a trend towards significance of *condition* (*χ*^2^(1) = 2.98, *p* = .084) (Figure 4b). on binary peeking behavior. Furthermore, there was a significant effect of *trial* (*χ*^2^(1) = 8.15, *p* = .005) on binary peeking behavior, with more peeking occurring in earlier trials (Figure 5 and Table S9). With regards to the exploration period as a proportion of entire trial duration over all trials, we found no significant effects of *condition* (*χ*^2^(1) = 0.59, *p* = .441) (Figure 6b) nor *trial* (*χ*^2^(1) = 0.60, *p* = .440) (Table S10) on the proportion peeking duration. Lastly, when analyzing overall binary occurrences of verbally asking ‘why?’, it was not appropriate to run a model as the question was asked by only two children a total of three times, all occasions occurring in non-functional conditions.

#### Exploratory Analyses.

In just the exploration period, we found significantly higher counts of peeking in the non-functional than functional condition (*χ*^2^(1) = 4.39, *p* = .036) and in earlier trials (*χ*^2^(1) = 7.11, *p* = .008) (Table S11). And when analyzing binary peeking behavior in the exploration time after the solve *in which the researcher was absent*, we found significant effects of both *condition* (*χ*^2^(1) = 4.46, *p* = .035) (Figure 4c) and *trial* (*χ*^2^(1) = 9.30, *p* = .002), with more peeking occurring in the non-functional than functional condition and in earlier trials (Table S12). When analyzing overall communication with the experimenter we found that children who communicated with the experimenter (*N* = 9) at all first did so in the non-functional condition (two-tailed binomial, *p* = 0.004). And regarding latency to the first peek, there were no significant effects of *condition* (*χ*^2^(1) = 0.28, *p* = .596) or *trial* (*χ*^2^(1) = 1.28, *p* = .257) (Table S13).

## DISCUSSION

We investigated epistemic curiosity in kea parrots and three-year-old human children following a violation-of-expectation event. Unlike the kea, the children peeked more often in the non-functional versus functional condition. The results also indicate that the children and not the kea peeked more often in earlier versus later trials, indicating effects of habituation on curiosity in the children. Importantly, however, 88% of kea and 48% of children peeked at least once, indicating that while there was no effect of condition, relatively more kea peeked than children overall. Put simply, the kea *were* curious, but indiscriminately between conditions.

A potential explanation for the differences in peeking between conditions in the two species could lie in how they value information (see Mettke-Hofmann et al., 2002). In the case of the kea, this highly neophilic parrot species is known not only for its extensive play and object manipulation (Diamond & Bond, 1999; Huber & Gajdon, 2006)—which could have predisposed subjects to interact with the apparatus regardless of condition—but also its interest in peeking. For example, a study by Schloegl et al. (2009) tasked kea with finding food hidden in one of two tubes, the location of which required peeking through the tubes’ openings. The authors found the kea were successful in identifying which tube contained the food, but commented on how redundant the subjects’ search behaviors were: Even after observing which tube contained food, the kea persisted in peeking not only in both straight tubes, but also both ends of both tubes, suggesting an intrinsic interest in the act of peeking that extended beyond locating food. In the present study, it is possible that either the kea valued peeking over the information gleaned from peeking, and/or the violation was simply not salient enough. We thus recommend future, relevant research in kea parrots incorporate a cost in gaining information (i.e., effortful peeking, see Range et al., 2009) and/or develop an independent measure for subjects’ sensitivity to the violating event to discern conditionally discriminate curious behaviors.

With regards to children’s increased epistemic curiosity in the non-functional compared to the functional condition this paper’s results are in-line with previous research. As discussed above, Stahl and Feigenson (2015) found that 11-month-old infants selectively explore objects that violated their expectations. Infants were more likely to drop objects that appeared to float horizontally off a shelf (and not fall to the ground due to gravity), as well as bang objects that appeared to pass through walls (rather than stop due to solidity). Furthermore, work by Gweon and Schulz (2011) found 16-month-old infants were more likely to pass a non-functional toy to a parent if they had seen it work before (failure of self), or try a third toy if they had observed a functional one and had been given a non-functional one (failure of toy). Our findings confirm that expectancy violations about the outcome of own actions on an apparatus are sufficient to epistemic curiosity seeking in preschool children.

A second potential explanation for the differences in peeking between the kea and children could be the subjects understanding of the apparatus. Importantly, during training, the children received verbal instruction regarding the apparatus. In contrast, such explanation was not possible to give to the kea. Instead, we trained subjects on the contingency between their actions (pushing in a block) and those of the apparatus (arm visibly moving and dropping the platform), and sought to examine whether a disruption of this contingency prompted peeking. However, future research may benefit from ensuring subjects understand the functionality of the apparatus, such as via less extensive training with the apparatus and/or greater access to the mechanism (i.e., arm) of the apparatus in the exploration period. Alternatively, a hint could be later provided as to how the apparatus was made non-functional to examine whether peeking decreases (see Baillargeon & Graber, 1987; Perez & Feigenson, 2022), and in fact, it is possible understanding the functionality of the apparatus in the children here contributed to their decreased peeking over trials.

It is also important to note the evident effect of researcher presence on the behavior of the children. Critically, we found that children were significantly more likely to peek in non-functional trials than functional trials while the researcher was absent, and any initial communication by a child to the researcher was also more likely in a non-functional trial (however, likelihood to peek as a factor of time passed was not considered). These results suggest an effect of adult presence on child exploration, which has also been seen in previous research. For example, work by Henderson (1984) suggests that adult familiarity influences child exploration, as some children are more likely to explore in the presence of a familiar (e.g., parent) rather than unfamiliar (e.g., researcher) adult. Considering this, it is important to note that in the present study, while the researcher left during the exploration period, the children were never left entirely alone: for those tested in museums, their parents were always within sight; and for those tested in schools, their teachers remained present. Thus, it is possible that some of the children in this study peeked more during the time in which the researcher left as it was while the unfamiliar adult was absent, and a familiar adult (parent or teacher) was still present (see also Tottenham et al., 2019). However, future work is needed to examine further how the presence of another person as well as their familiarity may influence exploration in children.

This finding is only relevant to the children in this experiment, however, as the researcher left during the thirty-second exploration only in the child experiment and not that of the kea (a methodological difference rooted in practicality, namely, avoiding opening and closing the encumbering aviary doors to allow for solo exploration). In the case of the kea, it is unclear whether we would have found any differences in behavior had the conditions been identical to those of the children, as previous research is mixed. For example, in Wein et al. (2018), kea did not change their likelihood to vocalize in the presence or absence of a researcher. Furthermore, research into bias in animal experiments suggests that factors such as human rearing and living with conspecifics—two relevant variables in the tested kea population—has a strong effect on expressed curiosity in animals (Damerius, Forss, et al., 2017; Damerius, Graber, et al., 2017; Schubiger et al., 2020). However, whether hand-reared kea living with conspecifics are more likely to express this curiosity in the absence or presence of a humans remains an open question. Based on the Wein et al. (2018) study as well as the authors’ experience testing the kea in this population, we did not suspect that the absence of a researcher would have affected peeking. While it is possible the human intervention (the real or fake placement of the peanut following the push of the wooden stick) could have disrupted information seeking, it was essential to provide the food in both conditions to thus make any information seeking non-conditional on the availability of the food reward. Importantly, however, we did not find a floor effect of information seeking, suggesting that human intervention was not an issue. Instead, we found that the kea showed more occurrences of peeking than the children, suggesting that the variable of food consumption similarly did not hinder their exploration within the time window. Overall, the kea did seek information in the test trials (despite the presence of the experimenter), but indiscriminately between the functional and non-functional condition. We therefore suggest that future research is needed to directly examine the effect of human presence on parrot exploration, in kea in particular.

Overall, while these two studies offer a refined approach to studying epistemic curiosity in humans and animals, they were not without limitations. For example, with regards to the kea, we suggest making peeking more costly and/or developing an independent measure of subjects’ sensitivity to the violation to ensure that peeking is, indeed, due to the violation of expectation. We also suggest exploring whether variables such as understanding apparatus functionality, apparatus exposure, as well as the role of social context are critical to expressed epistemic curiosity in kea or children following a violation-of-expectation event. Furthermore, to make inferences about the evolution of curiosity in these species, a more comprehensive phylogenetic investigation is necessary to tease apart homology from analogy.

As described earlier, the interpretation of previous epistemic curiosity research in animals suffers from methodological flaws such as withholding reward (Povinelli & Dunphy-Lelii, 2001), or not controlling for the saliencies of other experimental variables (e.g., object preference) (Demery, 2013). In summary, our results suggest that since the children and not the kea peeked more in the non-functional versus functional condition, children and not kea exhibited epistemic curiosity—the pursuit of information for its own sake—in this context.

## ACKNOWLEDGMENTS

We thank the kea keepers at Haidlhof Research Station as well as the Oakland Zoo, the Early Childhood Education Program at UC Berkeley and the SOMACC Yerba Buena Gardens Child Development Center for hosting the child research. Thanks also go to Wolfgang Berger and the UC Berkeley research technicians for the apparatuses, and Gemini Waterhouse, Rashika Singh and Sydney Tran for reliability coding. We also thank Macklin Hill, Julian Shea, and Anjuli Niyogi for their help with collecting the child data.

## FUNDING INFORMATION

This research was supported in part by the Austrian Science Fund (FWF, project P34533) grant to MLL.

## AUTHOR CONTRIBUTIONS

G.E.S.: Investigation (Equal); Writing – original draft (Lead); Writing – review & editing (Lead). M.L.L.: Conceptualization (Supporting); Funding acquisition (Lead); Resources (Lead); Supervision (Lead); Writing – original draft (Supporting); Writing – review & editing (Supporting). E.S.: Investigation (Equal); Writing – original draft (Supporting); Writing – review & editing (Supporting). J.M.E.: Conceptualization (Lead); Investigation (Supporting); Methodology (Lead); Resources (Lead); Supervision (Lead); Validation (Lead); Writing – original draft (Supporting); Writing – review & editing (Supporting). C.J.V.: Conceptualization (Lead); Data curation (Lead); Formal analysis (Lead); Investigation (supporting); Methodology (Lead); Supervision (Supporting); Validation (Lead); Visualization (Lead); Writing – original draft (Lead); Writing – review & editing (Lead).

## DATA AVAILABILITY STATEMENT

A data file and supplemental material are available for download on the article’s webpage: https://doi.org/10.1162/OPMI.a.34.

## Supplementary Material





## References

[bib1] Ajuwon, V., Monteiro, T., Schnell, A. K., & Clayton, N. S. (2025). To know or not to know? Curiosity and the value of prospective information in animals. Learning & Behavior, 53(1), 114–127. 10.3758/s13420-024-00647-y, 39414697 PMC11880187

[bib2] Auersperg, A. M., von Bayern, A. M., Gajdon, G. K., Huber, L., & Kacelnik, A. (2011). Flexibility in problem solving and tool use of kea and New Caledonian crows in a multi access box paradigm. PLoS ONE, 6(6), e20231. 10.1371/journal.pone.0020231, 21687666 PMC3110758

[bib3] Baillargeon, R., & Graber, M. (1987). Where's the rabbit? 5.5-month-old infants' representation of the height of a hidden object. Cognitive Development, 2(4), 375–392. 10.1016/S0885-2014(87)80014-X

[bib4] Bates, D., Mächler, M., Bolker, B., & Walker, S. (2015). Fitting linear mixed-effects models using lme4. Journal of Statistical Software, 67(1), 1–48. 10.18637/jss.v067.i01

[bib5] Berlyne, D. E. (1949). ‘Interest’ as a psychological concept. British Journal of Psychology, 39(4), 184–195. 10.1111/j.2044-8295.1949.tb00219.x, 18147696

[bib6] Berlyne, D. E. (1950). Novelty and curiosity as determinants of exploratory behaviour. British Journal of Psychology, 41(1–2), 68–80. 10.1111/j.2044-8295.1950.tb00262.x

[bib7] Berlyne, D. E. (1954). A theory of human curiosity. British Journal of Psychology, 45(3), 180–191. 10.1111/j.2044-8295.1954.tb01243.x, 13190171

[bib8] Berlyne, D. E. (1960). Conflict, arousal, and curiosity. McGraw-Hill Book Company. 10.1037/11164-000

[bib9] Berlyne, D. E. (1966). Curiosity and exploration: Animals spend much of their time seeking stimuli whose significance raises problems for psychology. Science, 153(3731), 25–33. 10.1126/science.153.3731.25, 5328120

[bib10] Brooks, M. E., Kristensen, K., van Benthem, K. J., Magnusson, A., Berg, C. W., Nielsen, A., Skaug, H. J., Machler, M., & Bolker, B. M. (2017). glmmTMB balances speed and flexibility among packages for zero-inflated generalized linear mixed modeling. R Journal, 9(2), 378–400. 10.32614/RJ-2017-066

[bib48] Cannon, W. B. (1932). The wisdom of the body. W. W. Norton & Company, Inc.

[bib11] Coleman, K., & Wilson, D. S. (1998). Shyness and boldness in pumpkinseed sunfish: Individual differences are context-specific. Animal Behaviour, 56(4), 927–936. 10.1006/anbe.1998.0852, 9790704

[bib12] Corey, D. T. (1978). The determinants of exploration and neophobia. Neuroscience & Biobehavioral Reviews, 2(4), 235–253. 10.1016/0149-7634(78)90033-7

[bib13] Damerius, L. A., Forss, S. I. F., Kosonen, Z. K., Willems, E. P., Burkart, J. M., Call, J., Galdikas, B. M. F., Liebal, K., Haun, D. B. M., & van Schaik, C. P. (2017). Orientation toward humans predicts cognitive performance in orang-utans. Scientific Reports, 7(1), 40052. 10.1038/srep40052, 28067260 PMC5220318

[bib14] Damerius, L. A., Graber, S. M., Willems, E. P., & van Schaik, C. P. (2017). Curiosity boosts orang-utan problem-solving ability. Animal Behaviour, 134, 57–70. 10.1016/j.anbehav.2017.10.005

[bib15] Day, H. I. (1971). The measurement of specific curiosity. In H. I. Day, D. E. Berlyne, & D. E. Hunt (Eds.), Intrinsic motivation: A new direction in education (pp. 99–112). Holt, Rinehart & Winston of Canada.

[bib16] Dember, W. N. (1956). Response by the rat to environmental change. Journal of Comparative and Physiological Psychology, 49(1), 93–95. 10.1037/h0045411, 13295416

[bib17] Demery, Z. P. (2013). Comparative sensory & cognitive adaptations for exploratory learning in parrots & humans [Doctoral dissertation]. University of Birmingham.

[bib18] Diamond, J., & Bond, A. B. (1999). Kea, bird of paradox: The evolution and behavior of a New Zealand parrot. University of California Press. 10.1525/9780520920804

[bib19] Forss, S., Ciria, A., Clark, F., Galusca, C.-L., Harrison, D., & Lee, S. (2024). A transdisciplinary view on curiosity beyond linguistic humans: Animals, infants, and artificial intelligence. Biological Reviews, 99(3), 979–998. 10.1111/brv.13054, 38287201

[bib20] Forss, S. I. F., Motes-Rodrigo, A., Dongre, P., Mohr, T., & van de Waal, E. (2022). Captivity and habituation to humans raise curiosity in vervet monkeys. Animal Cognition, 25(3), 671–682. 10.1007/s10071-021-01589-y, 34855018 PMC9107434

[bib21] Gajdon, G. K., Ortner, T. M., Wolf, C. C., & Huber, L. (2013). How to solve a mechanical problem: The relevance of visible and unobservable functionality for kea. Animal Cognition, 16(3), 483–492. 10.1007/s10071-012-0588-5, 23269471

[bib22] Gweon, H., & Schulz, L. (2011). 16-month-olds rationally infer causes of failed actions. Science, 332(6037), 1524. 10.1126/science.1204493, 21700866

[bib23] Henderson, B. B. (1984). Parents and exploration: The effect of context on individual differences in exploratory behavior. Child Development, 55(4), 1237–1245. 10.2307/1129993

[bib24] Huber, L., & Gajdon, G. K. (2006). Technical intelligence in animals: The kea model. Animal Cognition, 9(4), 295–305. 10.1007/s10071-006-0033-8, 16909237

[bib25] James, W. (1890). The principles of psychology (Vol. 1). Henry Holt & Co. 10.1037/10538-000

[bib26] Kidd, C., & Hayden, B. Y. (2015). The psychology and neuroscience of curiosity. Neuron, 88(3), 449–460. 10.1016/j.neuron.2015.09.010, 26539887 PMC4635443

[bib27] Kumar, S., Suleski, M., Craig, J. M., Kasprowicz, A. E., Sanderford, M., Li, M., Stecher, G., & Hedges, S. B. (2022). TimeTree 5: An expanded resource for species divergence times. Molecular Biology and Evolution, 39(8), msac174. 10.1093/molbev/msac174, 35932227 PMC9400175

[bib28] Lambert, M. L., Schiestl, M., Schwing, R., Taylor, A. H., Gajdon, G. K., Slocombe, K. E., & Seed, A. M. (2017). Function and flexibility of object exploration in kea and New Caledonian crows. Royal Society Open Science, 4(9), 170652. 10.1098/rsos.170652, 28989768 PMC5627108

[bib29] Liquin, E. G., & Lombrozo, T. (2020). Explanation-seeking curiosity in childhood. Current Opinion in Behavioral Sciences, 35, 14–20. 10.1016/j.cobeha.2020.05.012

[bib30] Loewenstein, G. (1994). The psychology of curiosity: A review and reinterpretation. Psychological Bulletin, 116(1), 75–98. 10.1037/0033-2909.116.1.75

[bib31] Markey, A., & Loewenstein, G. (2014). Curiosity. In R. Pekrun & L. Linnenbrink-Garcia (Eds.), International handbook of emotions in education (pp. 228–245). Routledge/Taylor & Francis Group.

[bib32] Mettke-Hofmann, C., Winkler, H., & Leisler, B. (2002). The significance of ecological factors for exploration and neophobia in parrots. Ethology, 108(3), 249–272. 10.1046/j.1439-0310.2002.00773.x

[bib33] Perez, J., & Feigenson, L. (2022). Violations of expectation trigger infants to search for explanations. Cognition, 218, 104942. 10.1016/j.cognition.2021.104942, 34740084

[bib34] Pisula, W. (2009). Curiosity and information seeking in animal and human behavior. Brown Walker Press.

[bib49] Poli, F., O’Reilly, J. X., Mars, R. B., & Hunnius, S. (2024). Curiosity and the dynamics of optimal exploration. Trends in Cognitive Sciences, 28(5), 441–453. 10.1016/j.tics.2024.02.001, 38413257

[bib35] Povinelli, D. J., & Dunphy-Lelii, S. (2001). Do chimpanzees seek explanations? Preliminary comparative investigations. Canadian Journal of Experimental Psychology / Revue Canadienne de Psychologie Expérimentale, 55(2), 185–193. 10.1037/h0087365, 11433789

[bib36] R Core Team. (2022). R: A language and environment for statistical computing. R Foundation for Statistical Computing. https://www.R-project.org/

[bib37] Range, F., Horn, L., Bugnyar, T., Gajdon, G. K., & Huber, L. (2009). Social attention in keas, dogs, and human children. Animal Cognition, 12(1), 181–192. 10.1007/s10071-008-0181-0, 18716802 PMC4415148

[bib38] Rosellini, R. A., & Widman, D. R. (1989). Prior exposure to stress reduces the diversity of exploratory behavior of novel objects in the rat (*Rattus norvegicus*). Journal of Comparative Psychology, 103(4), 339–346. 10.1037/0735-7036.103.4.339, 2598620

[bib39] Schloegl, C., Dierks, A., Gajdon, G. K., Huber, L., Kotrschal, K., & Bugnyar, T. (2009). What you see is what you get? Exclusion performances in ravens and keas. PLoS ONE, 4(8), e6368. 10.1371/journal.pone.0006368, 19654864 PMC2715862

[bib40] Schubiger, M. N., Fichtel, C., & Burkart, J. M. (2020). Validity of cognitive tests for non-human animals: Pitfalls and prospects. Frontiers in Psychology, 11, 1835. 10.3389/fpsyg.2020.01835, 32982822 PMC7488350

[bib41] Schuppli, C., Nellissen, L., Carvajal, L., Ashbury, A. M., Oliver-Caldwell, N., Rahmaeti, T., Laumer, I., & Haun, D. (2023). Ecological, social, and intrinsic factors affecting wild orangutans’ curiosity, assessed using a field experiment. Scientific Reports, 13(1), 13184. 10.1038/s41598-023-39214-2, 37580333 PMC10425418

[bib42] Smithson, M., & Verkuilen, J. (2006). A better lemon squeezer? Maximum-likelihood regression with beta-distributed dependent variables. Psychological Methods, 11(1), 54–71. 10.1037/1082-989X.11.1.54, 16594767

[bib43] Stahl, A. E., & Feigenson, L. (2015). Observing the unexpected enhances infants’ learning and exploration. Science, 348(6230), 91–94. 10.1126/science.aaa3799, 25838378 PMC5861377

[bib44] Tottenham, N., Shapiro, M., Flannery, J., Caldera, C., & Sullivan, R. M. (2019). Parental presence switches avoidance to attraction learning in children. Nature Human Behaviour, 3(10), 1070–1077. 10.1038/s41562-019-0656-9, 31332302 PMC7218758

[bib45] Völter, C. J., Lambert, M. L., & Huber, L. (2020). Do nonhumans seek explanations? Animal Behavior and Cognition, 7(3), 445–451. 10.26451/abc.07.03.10.2020, 39044805 PMC7616290

[bib46] Völter, C. J., Tomašić, A., Nipperdey, L., & Huber, L. (2023). Dogs’ expectations about occlusion events: From expectancy violation to exploration. Proceedings of the Royal Society B, 290(2003), 20230696. 10.1098/rspb.2023.0696, 37464755 PMC10354481

[bib47] Wein, A., Schwing, R., Hausberger, M., Rodriguez, R., & Huber, L. (2018). Vocal conditioning in kea parrots (*Nestor notabilis*). Journal of Comparative Psychology, 132(1), 97–105. 10.1037/com0000098, 29283587

