# Supplementary material for: Epistemic Curiosity in Kea Parrots and Human Children
Source: Open Mind (Camb). 2025 Sep 17;9:1528–42. doi: 10.1162/OPMI.a.34 (PMC12506928; doi:10.1162/OPMI.a.34)
Supplement: Supplementary file 1 [file opmi-09-1528-s001.pdf]

SUPPLEMENTARY MATERIALS

EXPERIMENT 1. KEA

Training

Traditional shaping methods of the kea resembled operant conditioning. Specifically, parrots were rewarded with the common food reward of cat pellets for every incremental behavior relevant to solving the task. In the beginning, touching the tool was rewarded with a pellet, as well as any pushing behavior of the tool. Following consistency in these behaviors, only pushing actions strong enough to move the arm were rewarded, continuing this until the subject could swiftly push the tool in and access the peanut on the now-collapsed tray.

Birds could experience 1-2 training sessions per testing day, with training duration varying per bird. This variance depended on the bird's apparent competency of the task, and to avoid frustration in subjects struggling to learn, the experimenter used her best judgement of the bird's behavior to determine how long training lasted per bird per session.

Table S1.

Demographic data of kea subjects

Subject	Sex	Age (years)	Rearing history
Coco	F	16	hand
Fay	F	7	parent
Frowin	M	19	parent
Jean-Luc	M	8	hand
John	M	24	parent
Kermit	M	19	hand
Kiri	F	8	parent
Lilly	F	16	hand
Mali	F	9	parent
Odo	M	8	parent
Paul	M	13	parent
Pick	M	19	hand
Plume	F	16	hand
Roku	M	15	parent
Skipper	M	6	hand

Sunny	F	16	hand
Tai	F	5	parent

Table S2.
Output of the model for kea overall binary peeking in exploration period

	Estimate	SE	χ^2	p	95% CI	
**(Intercept)**	-1.15	0.41			-2.28	-0.38
**Condition_Non-functional**	0.03	0.39	0.01	0.942	-0.8	0.89
**Z.trial**	-0.21	0.16	1.67	0.197	-0.63	0.15
**Z.session**	-0.22	0.16	1.71	0.191	-0.63	0.09

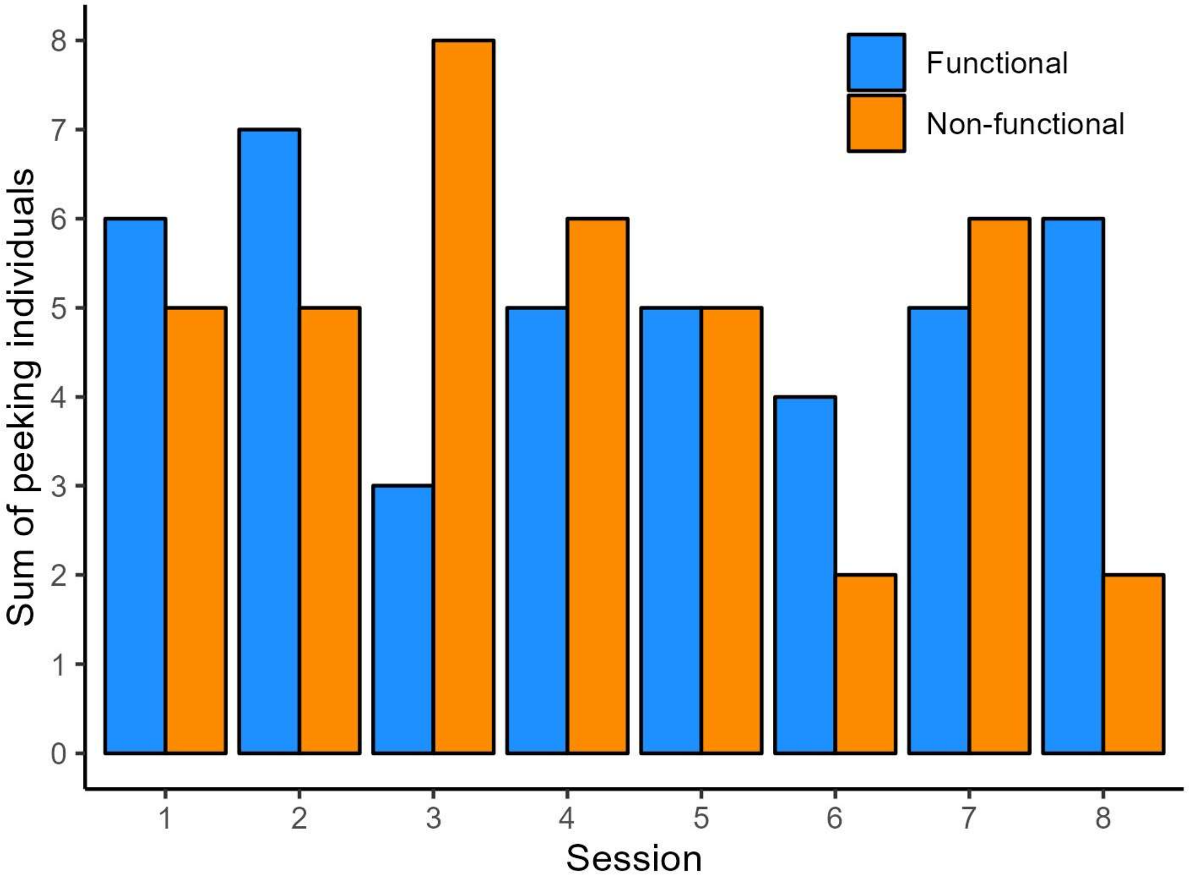

Figure S1. No effect of session was found on binary peeking behavior in kea.

Table S3.
Output of the model for kea peeking duration in exploration period as a proportion of entire trial duration over all trials

	Estimate	SE	χ^2	p	95% CI	
**(Intercept)**	-3.95	0.12			-4.2	-3.73

**Condition_Non-functional**	-0.02	0.11	0.02	0.887	-0.26	0.21
**Z.trial**	-0.06	0.06	1.1	0.295	-0.18	0.05
**Z.session**	-0.04	0.06	0.55	0.457	-0.16	0.07

Table S4.

Output of the model for kea binary peeking behavior in exploration period during the first session

	Estimate	SE	χ^2	p	95% CI	
**(Intercept)**	-2.07	1.38			-28.14	-0.21
**Condition_Non-functional**	-0.38	1.16	0.11	0.742	-35.1	2.95
**Order_Non-functional_first**	1.72	1.27	2.77	0.096	-0.18	28.07

Table S5.

Output of the model for kea peeking duration in exploration period as a proportion of entire trial duration in the first session

	Estimate	SE	χ^2	p	95% CI	
**(Intercept)**	-3.62	0.23			-4.12	-3.23
**Condition_Non-functional**	-0.07	0.22	0.11	0.743	-0.53	0.32
**Order_Non-functional_first**	0.33	0.24	1.93	0.165	-0.1	0.84

Table S6.

Output of the model for kea number of peeks per trial during exploration period

	Estimate	SE	χ^2	p	95% CI	
**(Intercept)**	-1.39	0.31			-2.21	-0.91
**Condition_Non-functional**	0.09	0.31	0.07	0.787	-0.56	0.76
**Z.trial**	-0.18	0.13	1.53	0.216	-0.5	0.09
**Z.session**	-0.2	0.14	1.92	0.166	-0.52	0.05

Note: No indication of overdispersion (dispersion parameter: 0.72)

Table S7.

Output of the model for kea latency to first peek in exploration period

	Estimate	SE	χ^2	p	95% CI	
**(Intercept)**	21.69	1.42			18.81	24.68
**Condition_Non-functional**	-1.62	1.96	0.66	0.418	-5.44	2.14
**Z.trial**	0.19	0.81	0.05	0.823	-1.61	1.85
**Z.session**	0.28	1.02	0.07	0.788	-1.8	2.27

EXPERIMENT 2. CHILDREN

Training

Children were shown a demonstration of the mechanism and a verbal instruction. The script proceeded as follows:

E1: point to sticker* “Look, here’s a prize. Let’s try to get it for you”.

“I’ll push this block. You see?” *push* “Look, now it fell down.” *touch reward collector*

“Can you put the prize in your prize box?” Box will be placed on the table, next to the apparatus

Table S8.

Demographic data of children subjects

Subject ID	Sex	Age (years and months)	Race
ES101	M	3y9m	African/African American
ES102	M	3y10m	White & American Indian or Alaskan Native
ES103	F	3y7m	Asian
ES104	F	3y2m	Asian
ES106	M	3y11m	Asian
ES108	M	3y2m	White
ES110	M	2y11m	White
ES111	F	3y2m	White
ES112	F	3y6m	Asian & White
ES113	F	3y4m	Hispanic/Latino
ES114	M	3y1m	Asian
ES115	F	2y8m	Asian
ES116	F	4y1m	Hispanic/Latino
ES117	F	3y1m	Asian
ES118	M	3y10m	White & Hispanic/Latino
ES119	M	3y	Other
ES120	M	3y10m	Asian
ES122	F	3y10m	White
ES123	F	3y8m	Asian
ES124	M	3y1m	Hispanic/Latino
ES125	M	3y2m	White & Hispanic/Latino
ES126	M	3y5m	Hispanic/Latino
ES127	M	3y1m	White
ES128	F	3y11m	White
ES129	F	4y1m	Pacific Islander
ES130	F	3y1m	Asian
ES132	F	3y0m	Asian
ES133	F	3y3m	Pacific Islander
ES134	F	3y6m	Asian
ES135	M	3y3m	Asian
ES136	F	3y8m	Asian

Table S9.

Output of the model for children overall binary peeking in exploration period

	**Estimate**	**SE**	**χ^2**	**p**	**95% CI**	
**(Intercept)**	-8.28	1.32			-16.74	-7.00
**Condition_Non-functional**	1.43	0.78	2.98	0.084	-1.56	7.16

**Z.trial**	-1.73	0.87	8.01	0.005	-4.29	-0.65

Table S10.

Output of the model for children peeking duration during exploration period as a proportion of entire trial duration over all trials

	Estimate	SE	χ^2	p	95% CI	
**(Intercept)**	-3.56	0.13			-3.85	-3.34
**Condition_Non-functional**	0.1	0.13	0.59	0.441	-0.16	0.37
**Z.trial**	-0.05	0.07	0.60	0.440	-0.18	0.08

Table S11.

Output of the model for children overall number of peeks per trial during exploration period

	Estimate	SE	χ^2	p	95% CI	
**(Intercept)**	-7.48	0.88			-10.47	-6.41
**Condition_Non-functional**	1.31	0.62	4.39	0.036	0.12	3.84
**Z.trial**	-1.05	0.54	7.11	0.008	-2.18	-0.28

Note: No indication of overdispersion (dispersion parameter: 0.48)

Table S12.

Output of the model for children overall binary peeking during exploration period with researcher absent

	Estimate	SE	χ^2	p	95% CI	
**(Intercept)**	-7.66	1.04			-16.01	-6.47
**Condition_Non-functional**	1.47	0.65	4.46	0.035	0.13	6.93
**Z.trial**	-1.48	0.66	9.3	0.002	-3.76	-0.48

Table S13.

Output of the model for children latency to peek during exploration period

	Estimate	SE	χ^2	p	95% CI	
**(Intercept)**	28.15	3.01			21.60	34.39
**Condition_Non-functional**	-2.55	4.19	0.28	0.596	-11.20	6.30
**Z.trial**	-3.01	2.52	1.28	0.257	-8.07	1.98
